# Arginase strongly impairs neuronal nitric oxide-mediated airway smooth muscle relaxation in allergic asthma

**DOI:** 10.1186/1465-9921-7-6

**Published:** 2006-01-12

**Authors:** Harm Maarsingh, John Leusink, I Sophie T Bos, Johan Zaagsma, Herman Meurs

**Affiliations:** 1 Department of Molecular Pharmacology, University Centre for Pharmacy, University of Groningen, Antonius Deusinglaan 1, 9713 AV Groningen, The Netherlands

## Abstract

**Background:**

Using guinea pig tracheal preparations, we have recently shown that endogenous arginase activity attenuates inhibitory nonadrenergic noncholinergic (iNANC) nerve-mediated airway smooth muscle relaxation by reducing nitric oxide (NO) production – due to competition with neuronal NO-synthase (nNOS) for the common substrate, L-arginine. Furthermore, in a guinea pig model of allergic asthma, airway arginase activity is markedly increased after the early asthmatic reaction (EAR), leading to deficiency of agonist-induced, epithelium-derived NO and subsequent airway hyperreactivity.

In this study, we investigated whether increased arginase activity after the EAR affects iNANC nerve-derived NO production and airway smooth muscle relaxation.

**Methods:**

Electrical field stimulation (EFS; 150 mA, 4 ms, 4 s, 0.5 – 16 Hz)-induced relaxation was measured in tracheal open-ring preparations precontracted to 30% with histamine in the presence of 1 μM atropine and 3 μM indomethacin. The contribution of NO to EFS-induced relaxation was assessed by the nonselective NOS inhibitor N^ω^-nitro-L-arginine (L-NNA, 100 μM), while the involvement of arginase activity in the regulation of EFS-induced NO production and relaxation was investigated by the effect of the specific arginase inhibitor N^ω^-hydroxy-nor-L-arginine (nor-NOHA, 10 μM). Furthermore, the role of substrate availability to nNOS was measured in the presence of exogenous L-arginine (5.0 mM).

**Results:**

At 6 h after ovalbumin-challenge (after the EAR), EFS-induced relaxation (ranging from 3.2 ± 1.1% at 0.5 Hz to 58.5 ± 2.2% at 16 Hz) was significantly decreased compared to unchallenged controls (7.1 ± 0.8% to 75.8 ± 0.7%; *P *< 0.05 all). In contrast to unchallenged controls, the NOS inhibitor L-NNA did not affect EFS-induced relaxation after allergen challenge, indicating that NO deficiency underlies the impaired relaxation. Remarkably, the specific arginase inhibitor nor-NOHA normalized the impaired relaxation to unchallenged control (*P *< 0.05 all), which effect was inhibited by L-NNA (*P *< 0.01 all). Moreover, the effect of nor-NOHA was mimicked by exogenous L-arginine.

**Conclusion:**

The results clearly demonstrate that increased arginase activity after the allergen-induced EAR contributes to a deficiency of iNANC nerve-derived NO and decreased airway smooth muscle relaxation, presumably via increased substrate competition with nNOS.

## Background

Nitric oxide (NO) is an important endogenous bronchodilator and is generated by a family of NO synthase (NOS) isoforms that utilize the semi-essential amino acid L-arginine, oxygen and NADPH as substrates to produce NO and L-citrulline [1]. In the airways, constitutive NOS (cNOS) isoforms – neuronal (nNOS) and endothelial NOS (eNOS) – are mainly expressed in inhibitory nonadrenergic noncholinergic (iNANC) neurons (nNOS), endothelium (eNOS) and epithelium (nNOS and eNOS), whereas inducible NOS (iNOS), which is induced by proinflammatory cytokines during airway inflammation, is mainly expressed in macrophages and epithelial cells [2].

Both in animal models and in patients it has been demonstrated that a deficiency of cNOS-derived NO is importantly involved in the development of airway hyperreactivity in allergic asthma [3-9]. Recent studies have indicated that alterations in L-arginine homeostasis play a major role in allergen-induced NO deficiency and airway hyperreactivity [10]. Thus, in a guinea pig model of allergic asthma we have demonstrated that a limitation of L-arginine to NOS underlies the allergen-induced NO-deficiency observed after the early asthmatic reaction (EAR) [11].

One mechanism that may be of particular importance in relation to reduced bioavailability of L-arginine in the airways is increased activity of arginase, which hydrolyzes L-arginine into L-ornithine and urea [10]. Arginase is expressed in the airways [12] and has shown to be functionally involved in the regulation of airway responsiveness to methacholine by competition with cNOS for the common substrate, L-arginine [13]. In a guinea pig model of allergic asthma, we have demonstrated that arginase activity in the airways is markedly increased after the allergen-induced EAR, thereby contributing to the observed NO-deficiency and subsequent airway hyperreactivity to methacholine [14]. Remarkably, inhibition of arginase activity by the specific inhibitor N^ω^-hydroxy-nor-L-arginine (nor-NOHA) completely reversed the airway hyperreactivity to the level of unchallenged controls by restoring NO production [14]. In line with these observations, increased arginase expression and/or activity have similarly been found in murine models of allergic asthma [15] and in asthmatic patients [15,16].

In a guinea pig model of asthma, a deficiency of cNOS-derived NO has previously also been implicated in reduced activity of the iNANC nervous system in the airways [17], which is the most effective bronchodilating neural pathway in both guinea pig and human airways [18-22]. Moreover, in guinea pig tracheal preparations we have recently demonstrated that endogenous arginase activity attenuates iNANC nerve-mediated NO production and airway smooth muscle relaxation under basal conditions, via competition with nNOS for L-arginine [23]. Therefore, in the present study we investigated the hypothesis that increased arginase activity in the airways induced by allergen challenge may also cause a deficiency of iNANC nerve-mediated NO and reduced airway smooth relaxation.

## Methods

### Animals

Male specified pathogen free Dunkin Hartley guinea pigs (Harlan Hillcrest, UK), weighing 500 – 800 g, were used. The animals were group-housed in individual cages in climate-controlled animal quarters and given water and food *ad libitum*, while a 12-h on/12-h off light cycle was maintained. The animals were IgE-sensitized to ovalbumin with Al(OH)_3 _as adjuvant as described [24] and used experimentally 4 to 6 weeks later.

All protocols described in this study were approved by the University of Groningen Committee for Animal Experimentation.

### Allergen provocation

Allergen provocations were performed by inhalation of an aerosolized solution of 0.5 mg/ml ovalbumin in saline as described [25]. Allergen inhalations were discontinued when the first signs of respiratory distress were observed. The animals were sacrificed 6 h after allergen provocation, *i.e*. after the EAR [26]. Nonchallenged animals served as controls.

### Tissue preparation

Guinea pigs were sacrificed by a sharp blow on the head followed by rapid exsanguination. The trachea was removed and prepared free of serosal connective tissue in a Krebs-Henseleit buffer solution of 37°C, gassed with 95% O_2 _and 5% CO_2_. The composition of the Krebs-Henseleit-solution in mM was: NaCl 117.50; KCl 5.60; MgSO_4 _1.18; CaCl_2 _2.50; NaH_2_PO_4 _1.28; NaHCO_3 _25.0 and D-glucose 5.50; pH 7.4. Twelve single proximal tracheal open-ring preparations were mounted for isotonic recording (0.3 g preload) between two parallel platinum point-electrodes in 20 ml organ baths containing gassed Krebs-Henseleit solution and indomethacin (3 μM) to eliminate any influence of prostanoids.

### Electrical field stimulation-induced relaxation experiments

After 30 min equilibration, tracheal preparations were relaxed with isoprenaline (0.1 μM) to establish basal tone. No difference in basal tone was observed in the preparations obtained from allergen challenged animals as compared to unchallenged animals. After thorough washout, maximal contraction to histamine was determined with cumulative additions of the agonist (0.1, 1, 10 and 100 μM). After washout, the preparations were precontracted with histamine to 30% of maximal histamine-induced tone in the presence of atropine (1 μM) to prevent electrical field stimulation (EFS)-induced cholinergic airway contraction. On the plateau, biphasic EFS (150 mA, 4 ms, 4 s, 0.5 – 16 Hz) was applied and one frequency-response curve (0.5 – 16 Hz in doubling steps) per preparation was recorded. When used, the nonselective NOS inhibitor N^ω^-nitro-L-arginine (L-NNA; 100 μM), the specific arginase inhibitor nor-NOHA (10 μM), a combination of the two inhibitors, or L-arginine (5.0 mM) were applied 30 min prior to histamine-administration. In line with previous observations [3,23], neither the NOS inhibitor, nor the arginase inhibitor and L-arginine affected agonist-induced contraction in the open-ring preparations. After the final EFS-induced relaxation, followed by washout, isoprenaline (10 μM) was added to re-establish basal tone. All measurements were performed in triplicate.

### Data analysis

All relaxations were estimated as peak height of the EFS-induced response, and were expressed as a percentage of maximal relaxation, as established in the presence of isoprenaline. The contribution of NO to the EFS-induced relaxation was determined by the effect of the NOS inhibitor L-NNA. Similarly, the role of arginase activity in the modulation of EFS-induced airway relaxation was determined by the effect of the arginase inhibitor nor-NOHA. The role of substrate availability in EFS-induced airway relaxation was assessed by measuring the response in the presence of exogenous L-arginine.

All data are expressed as means ± s.e.m. of *n *experiments. Statistical significance of differences was evaluated using a paired or unpaired two-tailed Student's t-test as appropriate, and significance was accepted when *P *< 0.05.

### Chemicals

Ovalbumin (grade III), aluminium hydroxide, histamine dihydrochloride, indomethacin, atropine sulphate, L-arginine hydrochloride, N^ω^-nitro-L-arginine and (-)-isoprenaline hydrochloride were obtained from Sigma Chemical Co. (St. Louis, MO, USA). N^ω^-hydroxy-nor-L-arginine was kindly provided by Dr J.-L. Boucher (Université Paris V, France).

## Results

**Figure 1:**
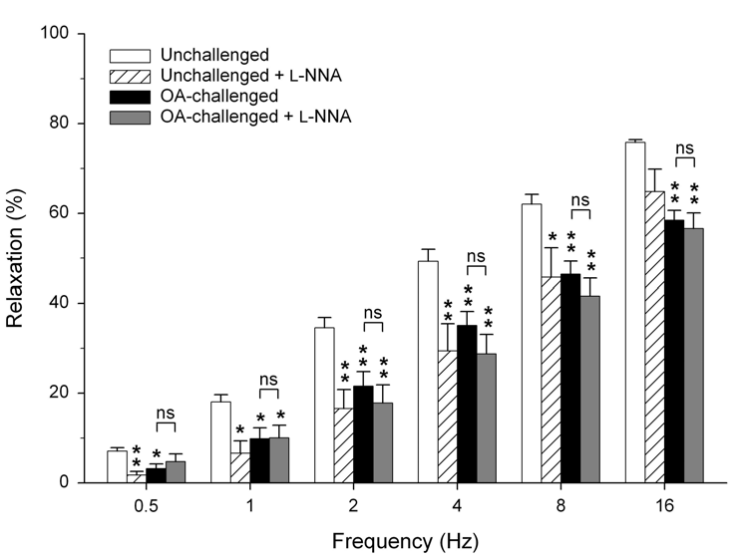
**iNANC nerve-induced airway smooth muscle relaxation is impaired after allergen challenge due to deficiency of nNOS-derived NO**. Electrical field stimulation-induced relaxation of precontracted tracheal open-ring preparations obtained from unchallenged and from OA-challenged guinea pigs in the absence and presence of the NOS inhibitor L-NNA (100 μM). Results are means ± s.e.m. of 7 experiments for each condition. *P < 0.05 and **P < 0.01 compared to unchallenged control, n.s. = non significant.

In line with our previous study [23], EFS (0.5 – 16 Hz) induced a frequency-dependent relaxation of histamine-induced tone of tracheal open-ring preparations from unchallenged guinea pigs, ranging from 7.1 ± 0.8% at 0.5 Hz to 75.8 ± 0.7% at 16 Hz. Incubation with the NOS inhibitor L-NNA (100 μM) caused a marked attenuation of the EFS-induced relaxation at 0.5 to 8 Hz, particularly at the lower frequencies (*P *< 0.05 all; Fig. 1).

At 6 h after ovalbumin-challenge (after the EAR), EFS-induced relaxations were significantly decreased at all frequencies as compared to unchallenged controls (values ranging from 3.2 ± 1.1% at 0.5 Hz to 58.5 ± 2.2% at 16 Hz; *P *< 0.05 all), to a similar extent as observed in L-NNA-treated preparations from unchallenged animals (Fig. 1). In contrast to tracheal preparations from unchallenged animals, L-NNA did not affect EFS-induced relaxations of the allergen-challenged preparations (Fig. 1), indicating deficiency of nNOS-derived NO after the allergen-induced EAR.

**Figure 2:**
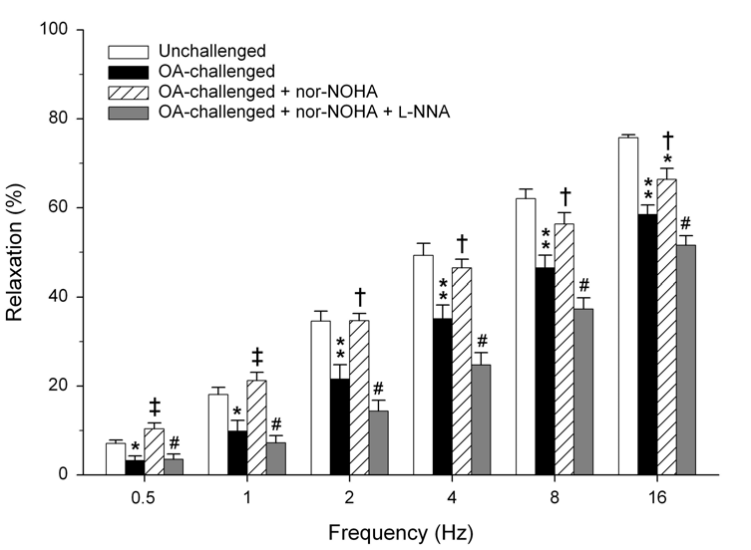
**Role of arginase and NO in impaired iNANC nerve-induced relaxation of guinea pig tracheal smooth muscle after allergen-challenge**. Electrical field stimulation-induced relaxation of precontracted tracheal open-ring preparations obtained from unchallenged and from OA-challenged guinea pigs in the absence and presence of the arginase inhibitor nor-NOHA (10 μM), with or without the NOS inhibitor L-NNA (100 μM). Results are means ± s.e.m. of 7 experiments for each condition. *P < 0.05 and **P < 0.01 compared to unchallenged control, ^†^*P *< 0.05 and ^‡^*P *< 0.01 compared to OA-challenged control, ^#^*P *< 0.01 compared to nor-NOHA-treated.

Interestingly, the impaired relaxations after allergen challenge were markedly increased by the specific arginase inhibitor nor-NOHA (10 μM) at all, but particularly the lower, frequencies (*P *< 0.05 all; Fig. 2). At 0.5 to 8 Hz, the iNANC relaxations were completely normalized to the level of unchallenged controls. The effect of the arginase inhibitor was reversed after coincubation with L-NNA at all frequencies (*P *< 0.01 all; Fig. 2).

**Figure 3:**
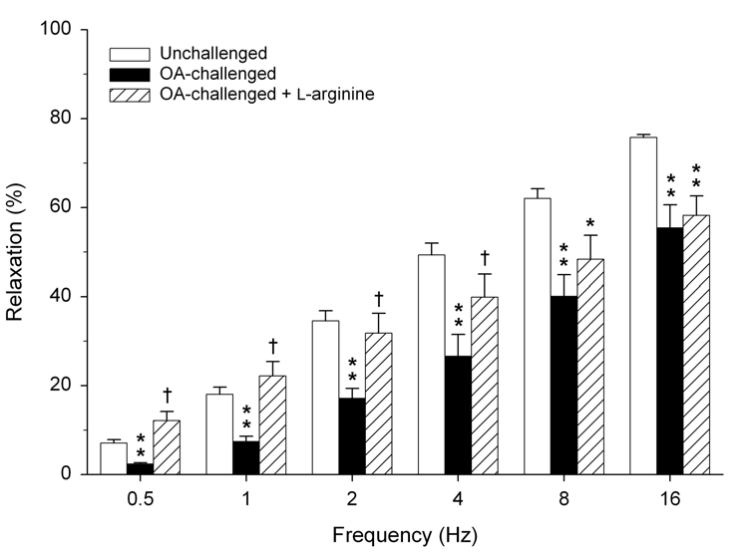
**Role of L-arginine availability in impaired iNANC nerve-induced relaxation of guinea pig tracheal smooth muscle after allergen-challenge**. Electrical field stimulation-induced relaxation of precontracted tracheal open-ring preparations obtained from unchallenged and from OA-challenged guinea pigs in the absence and presence of L-arginine (5.0 mM). Results are means ± s.e.m. of 4 (OA-challenged) to 7 (unchallenged) experiments. *P < 0.05 and **P < 0.01 compared to unchallenged control, ^†^*P *< 0.05 compared to OA-challenged control.

Similar to nor-NOHA, administration of exogenous L-arginine (5.0 mM) reversed the impaired relaxations after the EAR, also particularly at the lower frequencies (Fig. 3).

## Discussion

In both guinea pigs and humans, the iNANC nervous system is the most effective bronchodilating neural pathway of the airways, in which NO and vasoactive intestinal polypeptide act as neurotransmitters [18-22,27,28]. Recently, we have demonstrated that endogenous arginase activity is functionally involved in the regulation of iNANC nerve activity in the airway wall [23]. Thus, using guinea pig tracheal preparations, it was shown that arginase attenuates iNANC nerve-mediated airway smooth muscle relaxation specifically by inhibition of NO generation, presumably by limiting L-arginine availability to nNOS [23]. In a guinea pig model of allergic asthma, we have now shown that increased arginase activity, which is present in the airways after the allergen-induced EAR [14], may cause a considerably reduced iNANC relaxation of airway smooth muscle, due to deficiency of NO production. This reduced iNANC activity and deficiency of neuronal NO may contribute to the observed airway hyperreactivity in allergic asthma.

In line with previous studies [18,23], it was demonstrated that the NOS inhibitor L-NNA inhibited EFS-induced iNANC relaxations of guinea pig tracheal preparations. This inhibition was most pronounced at the lower frequencies (0.5 to 4 Hz), indicating a prominent role of nNOS-derived NO at these frequencies. Remarkably, allergen challenge resulted in reduced iNANC relaxations to the level of control preparations in the presence of L-NNA. Moreover, the reduced relaxations after allergen-challenge were not affected by L-NNA, indicating that a deficiency of nNOS-derived NO underlies the impaired iNANC relaxations.

Inhibition of arginase activity restored the impaired iNANC relaxations particularly at lower frequencies, indicating that increased arginase activity strongly restricts iNANC nerve-mediated airway smooth muscle relaxation. The increased relaxation after arginase inhibition was completely reversed by L-NNA, demonstrating that arginase activity attenuates iNANC nerve-mediated airway smooth muscle relaxation by limiting NO production. Since arginase activity in guinea pig airway preparations is markedly increased after the EAR [14], it seems obvious that increased competition with nNOS for L-arginine underlies the deficiency of nNOS-derived NO and subsequently impaired relaxations. That a limitation of endogenous L-arginine is involved indeed, was indicated by the effect of exogenous L-arginine, which, as nor-NOHA, normalized iNANC relaxations at the lower frequencies to the level of unchallenged controls. Using perfused tracheal preparations, we have previously demonstrated that L-arginine limitation due to increased arginase activity after the EAR also contributes to deficiency of agonist-induced, epithelium-derived NO and subsequent airway hyperreactivity [3,11,14]. Taken together, increased arginase activity upon allergen challenge causes a deficiency of both neural and nonneural cNOS-derived NO in the airways, which strongly contributes to the development of allergen-induced airway hyperreactivity after the EAR.

Although arginase is classically considered to be an enzyme of the urea cycle in the liver, it also occurs in many extrahepatic cells and tissues [29], where it may regulate NO synthesis by limiting the availability of intracellular L-arginine to NOS [12,29-31]. Two distinct isoforms of arginase have been identified: the cytosolic enzyme arginase I, highly expressed in the liver, and the mitochondrial arginase II, mainly expressed in extrahepatic tissues [29]. Both arginase isoforms are constitutively expressed in the airways, particularly in the bronchial epithelium and in fibroblasts from peribronchial connective tissue [12]. It has been demonstrated that the Th2 cytokines IL-4 and IL-13 increase arginase activity and expression of both isoforms in mouse lung [15] and in rat airway fibroblasts [32]. Increased arginase expression and activity was also found in a murine model of allergic asthma, both after challenge with ovalbumin and with *Aspergillus fumigatus*. Moreover, among the 291 common genes induced by these allergens, arginase I and II belonged to the most predominantly overexpressed genes [15]. Furthermore, in sensitized mice challenged with *Schistosoma mansoni *eggs increased arginase I gene expression was observed in wild type and Th2 polarized mice, but not in Th1 polarized animals [33].

The importance of arginase activity in the pathophysiology of allergic asthma has been indicated by the finding that arginase I protein expression is increased in bronchial alveolar lavage cells of asthmatic patients [15]. In addition, enhanced mRNA expression of arginase I was observed in the airway epithelium and in inflammatory cells in bronchial biopsies of these patients [15]. Morris and coworkers demonstrated that while plasma L-arginine levels are decreased in asthmatic patients during exacerbation, serum arginase activity is markedly increased [16], thus supporting the hypothesis that a disturbed L-arginine homeostasis due to increased arginase activity underlies airway hyperreactivity in allergic asthma [10].

A deficiency of iNANC nerve-derived NO has previously also been demonstrated by Miura *et al*. in sensitized guinea pigs airways at 24 h after the last of three consecutive daily ovalbumin-challenges. This could be restored in part by incubation with superoxide dismutase, indicating that scavenging of NO by superoxide could be involved [17]. In the present study, we demonstrated that NO-induced iNANC relaxation is already impaired at 6 h after single allergen-challenge. Using the same model, we have previously found that superoxide anions are not involved in the deficiency of nonneural, agonist-induced cNOS-derived NO at 6 h after single challenge [34], making its possible role in the reduced iNANC activity at this time-point unlikely.

In addition to arginase, downregulation of nNOS could also be involved in reduced iNANC activity. However, an effect of allergen challenge on the expression of nNOS was not found by Miura and coworkers [17]. Moreover, in the present study we demonstrated that increasing the availability of L-arginine – by arginase inhibition or exogenous addition of the amino acid – reversed impaired iNANC nerve-mediated airway smooth muscle relaxations, indicating that downregulation of nNOS does not underlie the impaired iNANC activity. This is line with previous studies, showing that nNOS expression is not affected by (repeated) allergen challenge [8,35,36], although some reduced expression of nNOS has been observed in one study [9].

Another mechanism which might contribute to L-arginine limitation after the EAR is impaired uptake of L-arginine caused by polycations. It has been reported that the major basic protein analogue poly-L-arginine inhibits L-arginine uptake [37] and causes airway hyperreactivity to methacholine in perfused guinea pig tracheal preparations by causing a deficiency of agonist-induced NO [38]. We have also demonstrated that endogenous (eosinophil-derived) polycations may be involved in allergen-induced NO deficiency and airway hyperreactivity after the EAR [39]. Thus, incubation with the polyanion heparin – acting as a scavenger of polycations – restored the production of NO and normalized airway reactivity to unchallenged control, presumably by restoration of cellular L-arginine uptake through cationic amino acid transporters [39].

Several studies have indicated that NO is the major neurotransmitter in iNANC nerve-mediated relaxations of guinea pig airways at lower frequencies [20,23,40,41], while EFS-induced relaxations at the higher frequencies may involve vasoactive intestinal polypeptide [20,41] as well as sympathetic nerve-derived noradrenaline [42]. This is in line with our observation that iNANC-mediated relaxations are more sensitive to L-NNA at the lower frequencies and very well explains why nor-NOHA is most effective at these frequencies.

A deficiency of iNANC nerve-derived NO has recently also been implicated in the pathophysiology of cystic fibrosis. Thus, in a mouse model of cystic fibrosis, impaired EFS-induced NO mediated airway smooth muscle relaxation was observed, which was reversed by L-arginine [43]. Moreover, it has recently been shown that arginase activity is increased in sputum obtained from patients with cystic fibrosis as compared to healthy controls [44]. In addition, a negative correlation between sputum arginase activity and levels of exhaled NO as well as FEV_1 _was observed in these patients [44]. A role for arginase by restricting the L-arginine availability to nNOS in iNANC nerves has also been proposed in the pathophysiology of erectile dysfunction [45]. In support, increased expression and activity of arginase II contributing to reduced iNANC nerve-derived NO production has been demonstrated in diabetic cavernosal tissue [46]. Moreover, neuronal arginase has shown to be involved in gastrointestinal motility disorders, by reducing nNOS-mediated iNANC relaxation in the internal anal sphincter [47].

## Conclusion

Using a guinea pig model of allergic asthma we have established that iNANC relaxations are markedly reduced after the allergen-induced EAR, due to a deficiency of nNOS-derived NO. Increased arginase activity is a major cause of the NO deficiency and impaired iNANC relaxation, presumably via increased substrate competition with nNOS.

## Abbreviations

cNOS, constitutive nitric oxide synthase; EAR, early asthmatic reaction; EFS, electrical field stimulation; eNOS, endothelial nitric oxide synthase; iNANC, inhibitory nonadrenergic noncholinergic; iNOS, inducible nitric oxide synthase; L-NNA, N^ω^-nitro-L-arginine; NADPH, nicotinamide adenine dinucleotide phosphate; nNOS, neuronal nitric oxide synthase; nor-NOHA, N^ω^-hydroxy-nor-L-arginine

## Competing interests

The author(s) declare that they have no competing interests.

## Authors' contributions

HMa designed and coordinated the study, performed part of the experiments, performed the statistical analysis and drafted the manuscript. JL performed part of the experiments. ISTB assisted substantially in performing the experiments. JZ participated in the design of the study, interpretation of results and final revision of the manuscript. HMe conceived of the study, participated in its design and direction, as well as in preparing the manuscript. All authors read and approved the final manuscript.
